# Dengue serosurvey after a 2-month long outbreak in Nîmes, France, 2015: was there more than met the eye?

**DOI:** 10.2807/1560-7917.ES.2018.23.23.1700482

**Published:** 2018-06-07

**Authors:** Tiphanie Succo, Harold Noël, Birgit Nikolay, Marianne Maquart, Amandine Cochet, Isabelle Leparc-Goffart, Olivier Catelinois, Henrik Salje, Camille Pelat, Perrine de Crouy-Chanel, Henriette de Valk, Simon Cauchemez, Cyril Rousseau

**Affiliations:** 1 The French Public Health Agency (Santé publique France), Regional unit (Cire) Occitanie, Saint-Maurice, France; 2 These authors contributed equally to the study and writing of the article; 3 The French Public Health Agency (Santé publique France), Saint-Maurice, France; 4 Mathematical Modelling of Infectious Diseases Unit, Institut Pasteur, Paris, France; 5 Centre National de la Recherche Scientifique, URA3012, Paris, France; 6 Center of Bioinformatics, Biostatistics and Integrative Biology, Institut Pasteur, Paris, France; 7 Institut de Recherche Biomédicale des Armées, National Reference Center for arboviruses, Marseille, France

**Keywords:** Dengue outbreak – Seroprevalence – Transmission risk – *Aedes albopictus* – Europe/France – West Nile virus

## Abstract

Clusters of dengue cases have recently become more frequent in areas of southern France colonised by the vector mosquito *Aedes albopictus*. In July 2015, a 2-month outbreak of dengue virus serotype 1 (DENV-1) was reported in Nîmes. **Aim:** We conducted a serosurvey in the affected area at the end of the vector activity period to determine the true extent of dengue transmission. **Methods:** We collected capillary blood from consenting household members, and information on their medical and travel histories, and exposure to mosquito bites. Recent infections were identified using IgM and IgG anti-DENV ELISA, followed, when positive, by plaque reduction neutralisation tests on serum against DENV 1–4 and West Nile virus. The prevalence estimator was calibrated on reference demographic data. We quantified the spatial clustering of dengue cases within the affected community and inferred the transmission tree. **Results:** The study participation rate was 39% (564/1,431). Three of 564 participants tested positive for DENV-1 infection (after marginal calibration, 0.41%; 95% confidence interval: 0.00–0.84). The spatial analysis showed that cases were clustered at the household level. Most participants perceived the presence of mosquitos as abundant (83%) and reported frequent mosquito bites (57%). We incidentally identified six past West Nile virus infections (0.9%; 95% CI: 0.2–1.6). **Conclusion:** This serosurvey confirms the potential for arboviral diseases to cause outbreaks − albeit limited for now − in France and Europe.

## Introduction

Dengue is the most common vector-borne viral disease worldwide, affecting 390 million people in tropical and sub-tropical areas each year [1]. The clinical spectrum of dengue ranges from a mild, non-specific febrile syndrome (classic dengue fever) to severe dengue with plasma leakage, haemorrhage or organ impairment [2]. In 20–97% of cases, dengue is clinically inapparent; this proportion varies considerably, across countries and years [3]. Asymptomatic infections may contribute significantly to virus transmission [4].

*Aedes aegypti* is the main vector of dengue virus in tropical areas [5]. Of more direct concern to continental Europe is a secondary vector, *Ae. albopictus*. Originally a tree-breeding mosquito of the forests of south-east Asia, *Ae. albopictus* has dramatically expanded its geographic distribution throughout the world in the past 40 years. Taking advantage of increasing global trade, *Ae. albopictus* spread to temperate regions in the late 1970s [6]. It was first reported in Europe in 1979 and has since been observed in France and 14 other countries along the Mediterranean. Given the recent establishment and rapid spread of *Ae. albopictus* in southern France, there is high potential for the emergence of arboviruses such as dengue virus (DENV), chikungunya and Zika viruses [7-10]. Limited local transmission of DENV was reported in southern France in 2010, 2013 and 2014, each cluster involving ≤ 2 cases [11-13]. Recently, from July to September 2015, a dengue outbreak occurred in the neighbourhood of Nîmes, Occitanie region [14]. Epidemiological investigations including door-to-door case finding conducted in August 2015 revealed seven autochthonous cases of dengue serotype 1 (DENV-1) that arose from a likely primary case over a 2-month period [14]. In France, a national surveillance and response plan aims to prevent and control local dissemination of dengue and other *Ae. albopictus*-transmitted viruses. However, little is known about the drivers and determinants of local transmission of dengue in the European setting, and the national response plan may not be fully effective.

In November 2015 we carried out a seroprevalence survey in order to determine the true extent of the dengue outbreak in Nîmes and the proportion of asymptomatic infections. We also reconstructed the transmission chain to characterise the spatial pattern of dengue transmission and to contribute to improving the performance of the French surveillance and response plan.

## Methods

### Study design

**Figure 1:**
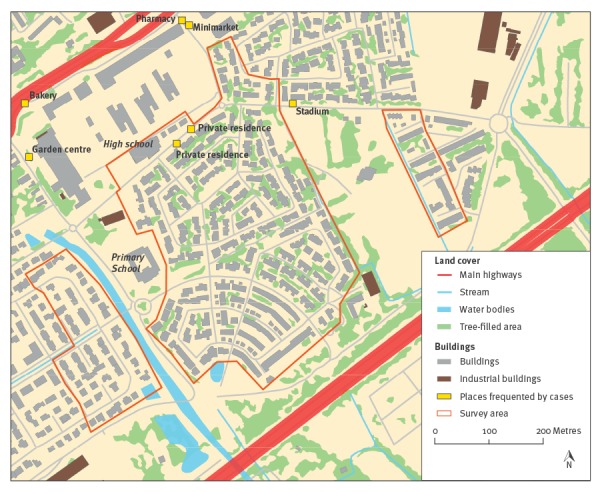
Map of the study area, dengue serosurvey, Nîmes, France, 2015

Source: Ign-BDTopo 2011; SOeS CORINE Land Cover 2015; Santé publique France, 2016.

We conducted a cross-sectional population-based serosurvey in the area affected by dengue in Nîmes. The study population included all members of households located within a 150 m radius around the residences of each case identified in the initial investigation. The study area covers the range where vector control measures were applied in compliance with the French preparedness and response plan [15]. In addition, the 109 households within a 100 m directly adjacent margin of the vector-control range were also eligible for inclusion (Figure 1). When > 50% of the houses in a block fell within the study area, we included the entire block. Inclusion criteria were: (i) residing in the study area since 1 July 2015; (ii) an age of 2 years or older. Patients under anticoagulant therapy were excluded. In total, 512 households were eligible, comprising an estimated 1,471 individuals of whom 1,431 were at least 2 years old (estimates obtained from the French National Institute of Statistics and Economic Studies (INSEE, ‘carroyages 200m’ and RFL2010, 2013); http://www.insee.fr/en/).

All eligible members of all households within the study area were invited to participate in the study. Enrolment took place between 6 and 20 November 2015, over a period of 9 days excluding holidays and weekends, from 09:00 to 15:00 and 15:00 to 21:00. For unresponsive households, we carried out three additional visits on separate dates and at different times of the day. After four visits without response, the household was considered non-respondent.

### Data and blood sample collection

Pairs of trained investigators, comprising at least one medical member of staff, interviewed the study participants, and collected capillary blood samples from fingertips on blotting paper (2–4 drops). They interviewed one adult per household (known henceforth as ‘the householder’) using a standardised questionnaire exploring household characteristics (address, demographics, vector-breeding sites, vegetation, protective measures such as window screens, air conditioning). In addition, the investigators interviewed each consenting household member (or parent for children) using a questionnaire to collect clinical data (medical history, symptoms compatible with dengue infection since 1 July, vaccination against flaviviruses), travel history and recent itineraries, individual risk factors for mosquito bites and dengue infection, knowledge and perception of the disease and its prevention.

### Laboratory analysis

Capillary blood samples were tested for serological evidence of dengue infection by in-house capture ELISA at the French National Reference Centre for Arboviruses (NRC) in Marseille [16]. Persons presenting IgM or IgG positive samples were requested to provide an additional blood sample by venepuncture, for plaque reduction neutralisation testing (PRNT) against DENV serotypes 1–4 and West Nile virus (WNV).

### Case definitions

A case of recent infection was defined as a person testing positive for anti-DENV IgM and IgG. Samples with isolated IgG and a PRNT positive for DENV (serotype 1 to 4) were classified as past infections.

A symptomatic case was defined as a case of recent infection who had presented with a febrile illness with body temperature ≥ 38 °C, with or without other symptoms, not explained by another medical condition, since 1 July 2015.

A case was classified as imported if the infected person had stayed in an area outside of continental France and Corsica, known at that time for being endemic or epidemic for dengue, in the 15 days preceding the date of onset of symptoms, or in the year before the interview for asymptomatic cases. Cases that did not meet the criteria of an imported case were classified as autochthonous.

The seven confirmed cases detected during the outbreak investigations conducted in August 2015 were all classified as autochthonous, recent dengue infections [14].

### Data analysis

#### Demographic characteristics

We compared the study participants with the reference population (i.e. the 1,431 inhabitants of the neighbourhood aged at least 2 years) in terms of age, sex and socio-professional category using the chi-squared test. We used nine age groups (2–3 years, 4–5 years, 6–10 years, 11–14 years, 15–17 years, 18–24 years, 25–64 years, 65–74 years and 75 years and older) and the eight categories defined by INSEE (farmer, tradesperson, upper white-collar, intermediate occupation, lower white-collar, blue-collar, retired, and never worked) [17]. The marginal distribution of these three variables in the reference population was calculated using information on two infra-communal areas covering the studied neighbourhood. These infra-communal areas, called IRIS (Ilots Regroupés pour l'Information Statistique) are grouped housing blocks created for the purpose of statistical information by INSEE [18].

#### Estimating prevalence of recent infections

Weights of the study participants, initially equal, were calibrated so that the estimated counts reflected the demographic structure of the reference population in terms of age structure and socio-professional category, as described above.

As the joint distribution of these two variables was not available, we applied a marginal calibration method using the marginal distributions obtained as specified in the previous paragraph. Specifically, we used the raking ratio method [19], implemented in the SAS macro CALMAR [20].

After calibration, the prevalence of recent dengue infections,$\hat{p}$,was estimated as follows:

$$\hat{p}=\frac{1}{N}\sum_{k=1}^{K}w_{k}$$

where *N* is the size of the reference population and $w_{k}$ the weights of the K participants who met the criteria for a case of recent dengue infection.

The variance of $\hat{p}$ was estimated as follows:

$$\hat{v}(\hat{p})=\left(1-\frac{n}{N}\right)\frac{\hat{p}(1-\hat{p})}{n-1}$$

where *n* is the size of the sample of participants. Of note, $\left(1-\frac{n}{N}\right)$ is called the finite population correction factor.

Finally, we calculated the 95% confidence interval (CI) as follows:

$$\text{95\% CI = }\left[\hat{p}\pm 1.96\sqrt{\hat{\nu }\left(\hat{p}\right)}\right]$$

We used SAS version 9.3 (SAS Institute, Cary, North Carolina, United States) to perform the analyses.

#### Description of the spatial patterns of observed cases and inferring the transmission tree

In this analysis, we used data from all cases identified through the seroprevalence survey and the seven confirmed cases identified through the outbreak investigation conducted in August 2015 [14]. Geographic coordinates of study participants’ residences were located using ArcGIS 10.2.2 software (ESRI Inc). Nine non-infected individuals with missing household coordinates were excluded from the analysis.

We calculated the median, minimum and maximum distances between pairs of cases and pairs of non-infected individuals based on their household locations. We quantified spatial clustering at various distance ranges in the community (same household, 0–50 m, >50–100 m and >100–200 m) as the Relative Risk (RR) of two cases living within a distance range relative to two non-infected individuals living within that distance range [21]. Details of the spatial clustering measure we used are provided in the Supplement. Confidence intervals (95% CI) of the RRs were estimated using normal approximation with small sample adjustment implemented in the epitools package in R.

We developed a statistical model to characterise the spread of DENV in this population and reconstructed the most likely transmission tree. The model jointly analysed data on DENV cases and non-infected individuals in the community and made it possible to test whether the rate of person-to-person transmission declined with distance. We assumed that the serial interval (i.e. time between symptom onset in a case and symptom onset in the persons they infected) of DENV was distributed with a mean of 16 days and a variance of 40 days (based on information about the different infection stages). Technical details are provided in the Supplement. We estimated parameters of the model using Bayesian Markov chain Monte Carlo sampling and report the posterior median with 95% credible intervals (95% CrI). We performed sensitivity analysis on the assumed serial interval distribution and we investigated the effect of potential case under-detection (Supplement).

#### Ethical approval

The study was carried out with the approval of the French Commission for Data Protection (Commission Nationale de l’Informatique et des Libertés). Participation was voluntary. Signed informed consent was obtained from all participants or their legal guardian for children.

## Results

### Survey participation and description of the participants

In total, 55% (282/512) of households and 39% (564/1,431) of residents participated in the study. The median age was 45 years (min–max: 2–86 years), with a male-to-female sex ratio of 0.80 (242/303). Of the participating householders 0.6% (3/542) were classified as farmers, 7% (36/542) as tradespeople, 2% (13/564) as upper white-collar, 16% (85/542) as intermediate occupation, 30% (165/542) as lower white-collar, 30% (164/542) as blue-collar, 13% (69/542) as retired and 1% (7/542) as never worked. The sample differed from the study population in terms of socio-professional categories (p < 0.0001) and age groups (p < 0.0001). Their sex distributions did not differ (p = 0.19). The categories of 18–24 years old, and lower white- or blue-collar were over-represented (Table 1).

**Table 1:** Distribution of age, sex and socio-professional category among study participants and reference populations, dengue serosurvey, Nîmes, France, 2015

	Participants		Reference population	
**Age group** **(years)**	**Number**	**Percentage** **(%)**	**Number**	**Percentage** **(%)**
2–3	8	1.4	20	1.4
4–5	5	0.9^a^	38	2.7
6–10	33	5.9	104	7.3
11–14	27	4.8 ^a^	109	7.6
15–17	13	2.3 ^b^	73	5.1
18–24	56	10.0 ^c^	53	3.7
25–64	352	62.9	887	62.0
65–74	46	8.2	110	7.7
≥ 75	20	3.6	37	2.6
**Total**	**564**	**100.0**	**1,431**	**100.0**
**Sex**				
Males	256	45.5	697	48.7
**Socio-professional category of the householder**				
Farmer	3	0.6	10	0.7
Tradesperson	36	6.6	77	5.4
Upper white-collar	13	2.4 ^c^	183	12.8
Intermediate occupation	85	15.7	203	14.2
Lower white-collar	165	30.4 ^c^	227	15.9
Blue-collar	164	30.3 ^c^	294	20.6
Retired	69	12.7 ^c^	307	21.5
Never worked	7	1.3 ^c^	130	9.1
**Total**	**542**	**100.0**	**1,431**	**100.0**

The reference population was estimated for the 512 households eligible for study participation based on data from the French National Institute of Statistics and Economic Studies (INSEE, ‘carroyages 200 m’ and RFL2010, 2013)

^a^ Proportion significantly different from the reference population (chi-squared test, p < 0.05).

^b^ Proportion significantly different from the reference population (chi-squared test, p < 10 ^− 2^).

^c^ Proportion significantly different from the reference population (chi-squared test, p < 10 ^− 4^).

A large proportion of participants reported an abundant presence of mosquitoes in the neighbourhood (83%) and frequent mosquito bites (57%) (Table 2. Most participants’ housing (98%) had a garden or terrace and its inhabitants frequently (88%) reported the presence of potential mosquito breeding sites (automatic watering, ornamental pond, temporary swimming pool, non-covered rainwater collection basin) (Table 2).

**Table 2:** Participants’ perception and behaviours regarding mosquito bites and their prevention, and household characteristics, dengue serosurvey, Nîmes, France, 2015

	Number	Percentage (%)
**Housing characteristics**		
Presence of a garden or terrace (n = 269)	264	98.1
Mosquito nets at windows (n = 280)	99	35.4
Use of air conditioning often or sometimes (n = 279)	91	32.6
Reported presence of mosquito breeding sites in the garden or terrace^a^ (n = 269)	237	88.1
Windows open during the day often or sometimes (n = 280)	155	55.4
**Perception and behaviours regarding mosquito bites and their prevention**		
Bitten by mosquito often or sometimes (n = 533)	304	57.0
Presence of mosquitoes perceived as very abundant or abundant (n = 533)	444	83.3
Use of insect repellent often or sometimes^b^ (n = 472)	248	52.5
Wearing long-sleeved shirts and long trousers often or sometimes^b^ (n = 468)	127	27.1

^a^ Automatic watering, ornamental pond, temporary swimming pool, non-covered rainwater collection basin.

^b^ Questions asked only of participants aged 15 years and older.

### Dengue seroprevalence and proportion of asymptomatic infections

**Figure 2:**
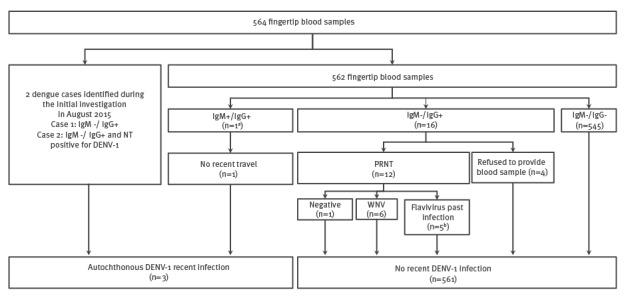
Biological results and classification of cases, dengue serosurvey, Nîmes, France, 2015

DENV: dengue virus; NT: neutralisation test; PRNT: plaque reduction neutralisation testing; WNV: West Nile virus ; +: positive; -: negative.

^a^ PRNT results: DENV recent infection of indeterminable serotype.

^b^ PRNT results: DENV past infection of indeterminable serotype (2 cases) or Flavivirus past infection (3 cases). All reported a history of travel in a dengue-endemic area. Six participants had IgG antibodies against WNV, confirmed by PRNT. We estimated the prevalence of past WNV infection at 0.9% (95% CI: 0.22–1.57).

The serosurvey identified three cases of recent autochthonous dengue infection: one tested positive for both anti-DENV IgM and IgG; two tested positive for anti-DENV IgG (these two had already been diagnosed during the outbreak investigation conducted in August 2015) (Figure 2). Of the five cases identified through the outbreak investigation, three cases were absent at the time of study implementation. Two cases in a household of five people refused to participate in the serosurvey on the grounds that they had taken part in the initial investigation. In total, eight autochthonous cases were identified across the two investigations. After calibration, dengue prevalence was estimated at 0.4% (95% CI, 0.0–0.8).

No asymptomatic case was identified. The three cases presented a febrile illness with body temperature ≥ 38 °C (n = 3), retro-orbital pain (n = 2), skin rash (n = 2), headache (n = 2), myalgia (n = 2), arthralgia (n = 2), digestive disorders (n = 1) and asthenia (n = 2).

### Spatial case clustering and reconstructed transmission tree

**Figure 3:**
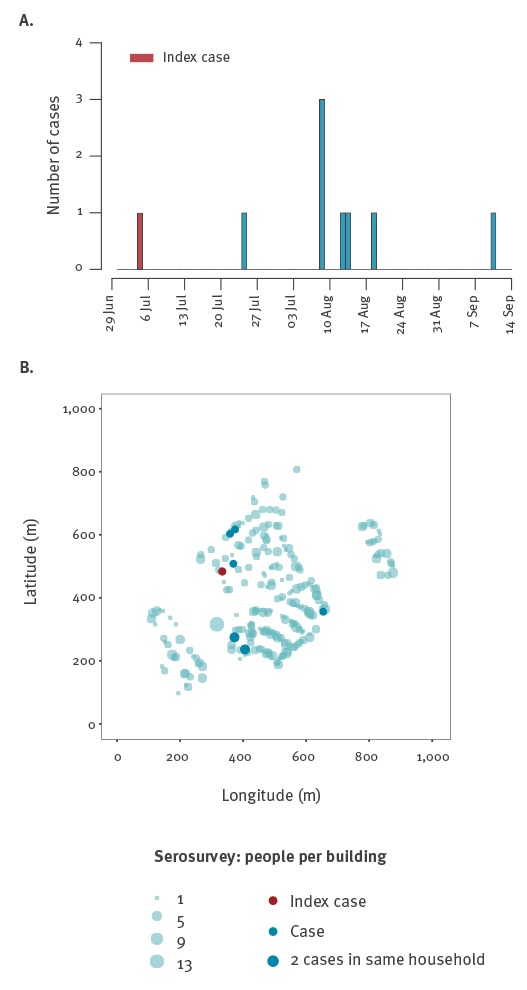
Epidemic curve (A), spatial distribution of cases (B), dengue serosurvey, Nîmes, France, 2015

**Figure 4:**
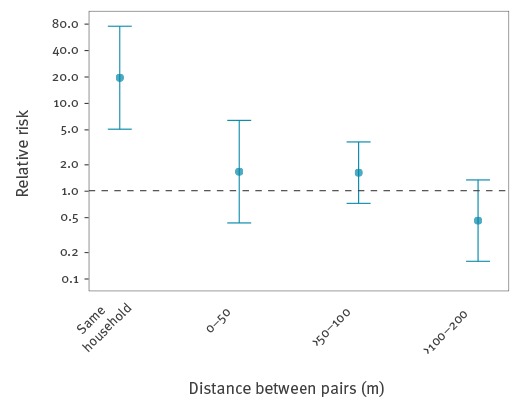
Spatial clustering of cases by household distance, dengue serosurvey, Nîmes, France, 2015

Following the importation of a likely primary case with disease onset on 6 July 2015, eight autochthonous cases developed illness within 68 days (Figure 3 A). The median distance between any two DENV cases (266 m; interquartile range (IQR): 106–333 m; range: 0–384 m) was similar to that observed between any two non-infected individuals recruited in the study (271 m; IQR: 155–385 m; range 0–816 m; Wilcoxon rank-sum test p = 0.064) (Figure 3 B). The spatial distribution of cases and non-infected individuals in the studied area suggested clustering of cases at the household level, where the risk of two DENV cases living in the same household was 18.2 (95% CrI: 4.7–70.2) times higher than the risk of two non-infected individuals living in the same household (Figure 4). There was no significant evidence for clustering at other distances in the study area of ca 800m^2^.

Spatial clustering was measured as the relative risk (RR) of two dengue virus cases living within a distance range compared to two non-infected individuals living within that distance range; where the risk of a pair being in that distance range was calculated as number pairs within the range/ number pairs within the range or further apart. A distance of 0 m represents individuals living in the same building but different households.

**Figure 5:**
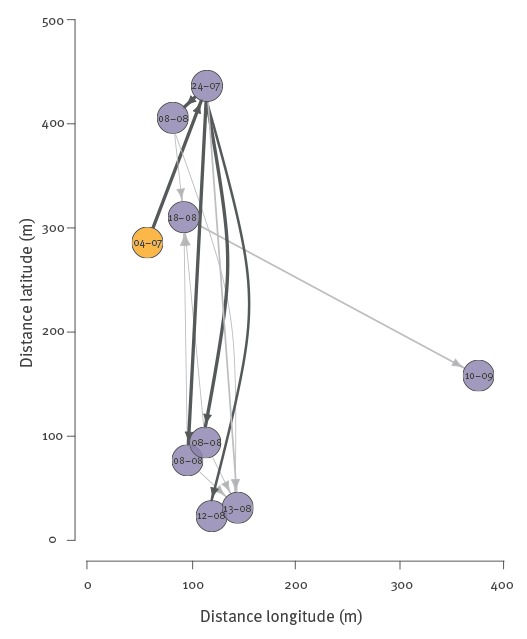
Reconstructed transmission tree, dengue serosurvey, Nîmes, France, 2015

We were able to identify the most likely infector (with > 50% probability) for five of the eight autochthonous cases. The reconstructed transmission tree is shown in Figure 5. The estimated effective reproduction number R (number secondary cases caused by a case) was 0.98 (95% CrI: 0.46–1.74). Cases from the same household most likely belonged to the same generation since their symptom onset dates were very close (0 or 1 day delay). We did not find evidence for a decline in the transmission rate by geographic distance from a case.

Shown are connections between cases with transmission probabilities ≥0.2; dark grey represents connections with probabilities ≥0.5. The node labels indicate the onset date of disease and node locations represent geographic household locations. Overlapping nodes represent cases who lived in the same household. The index case is indicated in red.

## Discussion

In order to determine the extent of the 2015 DENV-1 outbreak in Nîmes, we conducted a serosurvey that identified three recent autochthonous dengue cases, which, together with those already known, led to an estimated dengue seroprevalence of 0.4%. No asymptomatic infection was detected in the affected area. Dengue transmission by local *Ae. albopictus* mosquitoes remained limited overall to eight autochthonous cases clustered within households.

Similar low-seroprevalence estimates have been documented in other studies carried out in dengue-affected areas in high-income countries: 1% in 2004 in Texas, US, 3% in 2009 in Florida, US, and 0.6% in Croatia in 2012 [22-24].

However, our study is not devoid of limitations inherent to field investigations. We cannot exclude the possibility that additional cases went undetected, since only 55% of the households and 39% of the inhabitants of the neighbourhood participated in the study. But with only one additional case identified by the serosurvey and nearly all of the cases captured by the initial investigation, this should marginally affect our estimate of dengue prevalence in the study area. Study participants had a different age and socio-professional make-up than the reference population. Though only partially, we set out to address the effect of non-response by calibrating our prevalence estimator on reference demographic data. We could not include five cases identified through the outbreak investigation, nor the households to which they belonged. The resulting underestimation of the prevalence of dengue should have remained limited because they represented only a couple of households, surrounded by other dengue-free households.

Our study highlighted a decline in dengue antibody titre 3 months after the onset of fever. Indeed, anti-dengue IgM were undetectable for the two cases already confirmed in August 2015. Theoretically this may have led to misclassification of cases of recent DENV infection among people with anti-DENV IgG but without IgM. This could have been overcome to some extent by carrying out serosurveys sooner after an outbreak. However, we chose to conduct our study in November, in the late-activity season of *Ae. albopictus* in southern France, to capture any potential residual transmission [25]. Four years after the establishment of *Ae. albopictus*, vector density has become high enough for dengue transmission in this neighbourhood of Nîmes, mostly composed of small properties with gardens. High vector density was also corroborated by our study findings with high levels of mosquito nuisance reported by the participants.

Physical barriers in the building and landscape features that surrounded the outbreak area, such as large roads, a nearby high school campus and stadium might have contributed to preventing a wider spread of the virus. Vector-control measures applied in the outbreak area in August and September 2015 most likely played a role in breaking the transmission chain, although the exact impact cannot be measured.

Seroprevalence data on the fifth limited dengue cluster observed in France since 2010 suggest that French *Ae. albopictus* may not be able to sustain explosive DENV transmission. A small number of dengue outbreaks transmitted by *Ae. albopictus* have been described worldwide in the last decade (Hawaii 2001, Gabon 2007, China 2014, Japan 2014) [26-29]. In Nîmes, transmission of DENV remained limited despite a 2-month long circulation of the virus, from July to September 2015. However, with 160 cases, the 2014 dengue outbreak in Tokyo, Japan, demonstrated that more intensive transmission is possible even in temperate climates and that continued vigilance is needed in areas colonised by *Ae. albopictus* [29].

The spatial analysis showed that cases clustered at the household level. The two case-pairs in same households developed symptoms either on the same day or within one day; these household members therefore belonged most likely to the same generation of cases. The household clustering may be explained by the common exposure to an infected mosquito in the household. We did not find evidence for a spatial decline in the transmission hazard by distance from a case, estimated on the basis of household locations of cases and non-infected individuals. Several previous studies found evidence for small scale patterns in DENV transmission [30-32]. In our study, the lack of spatial transmission patterns may be due to the small number of observed DENV cases resulting in limited power to detect a significant decline in the transmission rate. The study area and the distances over which DENV cases occurred were also relatively small and spatial effects may have been more apparent if a wider area was investigated or affected. Moreover, though households are generally considered as foci for DENV transmission, individuals may have acquired infections at other locations than their homes.

No asymptomatic cases were identified through the outbreak investigation and serosurvey [14]. Most cases could reasonably be placed on a transmission tree reconstructed only on the basis of time and place of occurrence of symptomatic cases. This suggests that the contribution, if any, of undetected asymptomatic cases was possibly not crucial in this outbreak. All in all, the eight locally acquired cases related to this outbreak reported fever ≥ 38 °C (n = 8), retro-orbital pain (n = 4), skin rash (n = 5), headache (n = 7), myalgia (n = 4), arthralgia (n = 2), digestive disorders (n = 4) and asthenia (n = 6). That no asymptomatic case was identified was unexpected. In the literature, the proportion of inapparent infections varies widely, from 20% to 97% in endemic areas [3]. The active case finding conducted during the initial investigation in August 2015, enhanced awareness and easy access to healthcare in the community might have led us to detect and classify as ‘symptomatic’ any pauci-symptomatic case that would have gone unnoticed elsewhere. The wide clinical spectrum of dengue complicates the determination of the frequency of asymptomatic cases as mentioned in a review of the literature on the extent of inapparent DENV infections [3].

Some studies suggest an association between the initial immunity and the clinical expression with a higher frequency of symptomatic dengue among non-immune people [33,34]. However, the factors associated with the clinical expression of dengue have not been clearly determined. It remains possible that in our study the number of DENV cases was too low to detect asymptomatic cases.

Our results incidentally evidenced low-level circulation of WNV in the study population. A WNV serosurvey, carried out in 2000, presented similar results among blood donors living inside and near Camargue, a regional nature park located 50 km from Nîmes, where WNV re-emerged among horses earlier the same year [35]. Our estimate of 0.9% falls between that of the prevalence of antibodies against WNV in blood donors living in Camargue at 1% and in contiguous regions at 0.2%.

## Conclusion

Transmission of DENV during the 2015 outbreak in Nîmes remained of low intensity and clustered within households despite a high perceived density of *Ae. albopictus* and a 2-month long circulation period of the virus.

The potential for arboviral diseases to cause outbreaks in France should not be underestimated. The geographical distribution of *Ae. albopictus* is expanding steadily while climate change could increase the potential for outbreaks northward. The intensification of international travel increases the risk of DENV introduction in the naïve population of mainland France. Moreover the competence of *Ae. albopictus* to transmit other major arboviruses has been demonstrated, in the field, for chikungunya virus and, in the laboratory, for Zika virus

More broadly, this risk is present in several other European countries where new areas are colonised by *Ae. albopictus* each year.

Our study suggests that surveillance and early outbreak investigation can successfully determine the extent of dengue emergence. Further serosurveys should be conducted around future outbreaks to monitor the evolution of the transmission dynamics of arboviral diseases in Europe. These studies could also further document the proportion of unapparent arboviral infections and their contribution to transmission, with the ultimate aim of adapting the control measures around autochthonous transmission.
